# Intracardiac Thrombus Formation and Bilateral Pulmonary Embolisms in a Patient With Behcet's Disease While on Regular Infliximab Infusion: A Case Report

**DOI:** 10.7759/cureus.18592

**Published:** 2021-10-08

**Authors:** Mohammed A Miqdad, Abdullah Mohamad, Fawaz Ali, Mohammed Kawari, Salha Alboainain

**Affiliations:** 1 Department of Internal Medicine, Dr. Sulaiman Al Habib Medical Group, Khobar, SAU; 2 Department of Internal Medicine, California Institute of Behavioral Neurosciences & Psychology, Fairfield, USA; 3 Department of Internal Medicine, Unit of Hematology, Dr. Sulaiman Al Habib Medical Group, Khobar, SAU; 4 Department of Rheumatology, Dr. Sulaiman Al Habib Medical Group, Khobar, SAU

**Keywords:** infliximab, anticoagulation, immunosuppressants, intracardiac thrombus, pulmonary embolism, thromboembolism, behcet's disease

## Abstract

Vascular complications of Behcet'sdisease, including intracardiac thrombus formation, are one of the significant causes of mortality and morbidity in this population. Similar to other vasculitic disorders, Behcet's disease is primarily treated with immunosuppressants. While the benefit of adding anticoagulants in Behcet's disease with thromboembolism remains debatable, some literature encourages its use with concomitant intracardiac thrombus. Herewith, we present the case of a young male who was diagnosed with bilateral pulmonary embolism in addition to right ventricle intracardiac thrombus upon his scheduled dose of infliximab infusion. He was managed by adding azathioprine to his regimen together with oral prednisolone and warfarin with a target international normalized ratio of 2-3. This case report addresses the importance and outcome of early identification of Behcet's disease's vascular complications and immediate initiation of anticoagulation accordingly.

## Introduction

Behcet's disease (BD) is a multisystemic vasculitis disease that is mainly characterized by oral and genital ulcers in addition to skin and ocular lesions [1]. While BD manifestations can extend to gastrointestinal, neurological, and cardiovascular systems, cardiac involvement in BD is associated with poor outcomes [1]. BD's vascular complications are among the major causes of morbidity and mortality in affected populations, with incident rates reaching up to 40%, mainly in young males being affected more commonly [2]. Furthermore, the venous thrombosis events mainly involve the venous system in the lower limbs with rare pulmonary artery involvement [2]. Nonetheless, pulmonary artery thrombosis of BD is almost always associated with intracardiac thrombi [1].

According to the European League Against Rheumatism (EULAR), the mainstay of vascular BD treatment is immunosuppressive agents, resulting in a declined recurrence rate proportionally with the length of therapy, based on a retrospective study involving 936 BD patients [2,3]. However, anticoagulation might be considered in addition to immunosuppressive therapy when intracardiac thrombi coexist [4,5]. Here we present a case of a young male who presented with multiple pulmonary embolisms and right-sided intraventricular mural thrombi. He was treated with combinations of immunosuppressants (infliximab and azathioprine) and warfarin, which resulted in a complete resolution of intracardiac thrombus after four weeks.

## Case presentation

**Figure 1:**
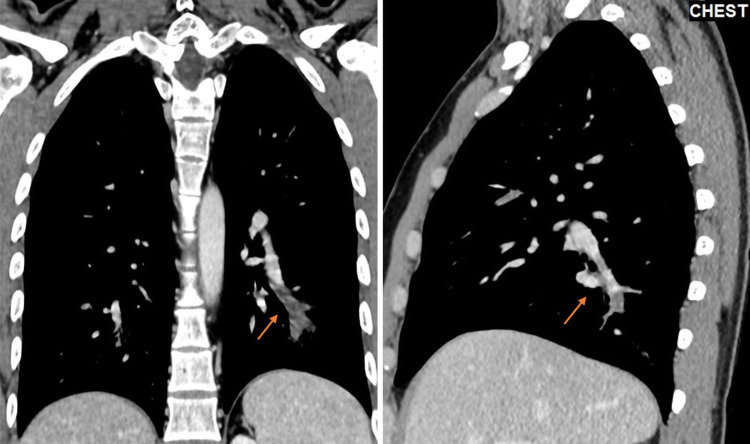
CAT scan of the chest with contrast Chest CAT scan with contrast showing filling defects involving multiple pulmonary artery branches on both sides, suggesting multiple bilateral pulmonary embolisms.

A 28-year-old male was diagnosed with BD in 2016 with no major organ involvement. He had a prolonged remitting course on oral steroids initially followed by scheduled infliximab infusion due to recurrent relapses in the shape of painful oral and genital ulcers. In June 2021, he was admitted electively to receive his scheduled infliximab dose. However, he was found to have a fever (38.4 °C) prior to infliximab administration, but other vital signs were within normal limits. Subsequently, infliximab was suspended, and routine blood workup together with chest X-ray was requested. Initial labs showed high inflammatory markers, including white blood count and C-reactive protein, and the chest X-ray revealed small faint opacity in the right lung. Therefore, a chest CAT scan with contrast was performed, which revealed filling defects involving multiple pulmonary artery branches on both sides, suggesting multiple bilateral pulmonary embolisms (Figure 1). 

**Figure 2:**
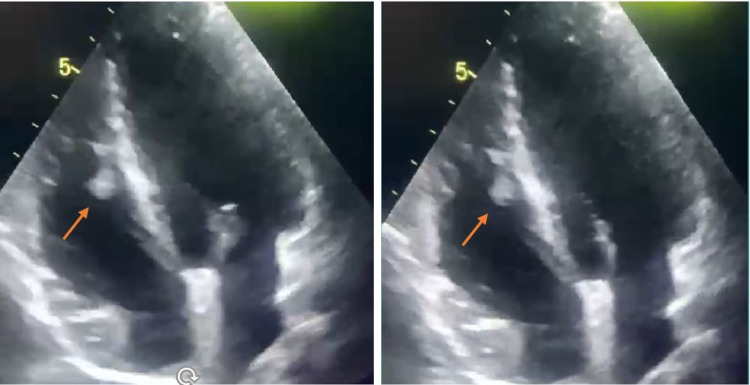
Echocardiography before anticoagulant initiation Transthoracic echocardiography showing mobile mural thrombus in the right ventricle.

**Figure 3:**
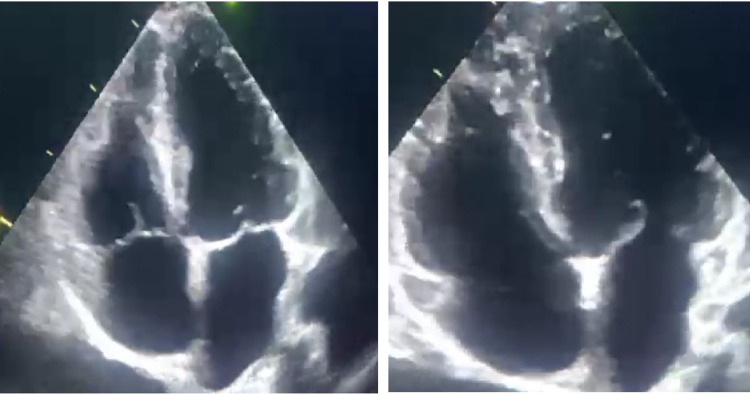
Echocardiography four weeks after anticoagulant initiation Transthoracic echocardiography showing complete resolution of the right ventricular thrombus after four weeks of anticoagulation.

Hence, he was immediately started on a therapeutic dose of enoxaparin along with supportive measures, such as paracetamol, upon necessity. Moreover, a transthoracic echocardiogram showed showering mural thrombus at the right ventricle (RV) (Figure 2). Further thrombophilia workup was ruled out, including factor V Leiden, homocysteine, anti-lupus anticoagulant, and anticardiolipin antibodies (Table 1). Consequently, a multidisciplinary team, including a rheumatologist, a cardiologist, and a hematologist, has decided to initiate anticoagulation (warfarin) with a target international normalized ratio of 2-3, oral prednisolone 0.5 mg/kg/day, and azathioprine in addition to his scheduled infliximab therapy. Four weeks later, follow-up echocardiography showed complete resolution of the intracardiac thrombus (Figure 3). Notably, warfarin was planned to be continued for six months, followed by prophylactic rivaroxaban 10 mg once daily lifelong. 

**Table 1:** Thromobiphilia workup ANA: anti-nuclear antibodies; dsDNA: double-stranded DNA.

Blood test	Result
Factor V Leiden	Negative
Anticardiolipin antibodies (IGG, IGM)	Negative
ANA	Negative
Anti dsDNA antibodies	Negative
Anti-lupus anticoagulant	Negative
Homocysteine	Normal

## Discussion

Arterial and venous thrombosis is the most frequent BD vascular complication, with a cardiac involvement of 1%-5% of clinical series [4]. Right-sided intracardiac thrombus formation (ICTF) is the most common frequently reported cardiac involvement, particularly in young males [5], as in our patient. The exact reason for the high frequency of right-sided involvement is not entirely understood yet, but overall, the thrombotic tendency in BD patients is mainly attributed to endothelial ischemic changes, which lead to activation of platelet aggregation [5]. Furthermore, BD vascular involvement was suggested to be related to neutrophils and thrombosis through nicotinamide adenine dinucleotide phosphate (NADPH) oxidase (specifically NOX2) by producing reactive oxygen species [6]. The latter can modify the secondary structure of fibrinogen, being less susceptible to plasmin-induced lysis [6]. Additionally, mean plasma homocysteine levels were significantly higher in BD patients than in healthy individuals [5].

Although the mainstay of treating vascular BD is immunosuppressant, the role of additional anticoagulation remains an area of controversy. However, a retrospective analysis of 37 patients with venous thrombosis in BD was performed [7]. These patients were categorized according to the course of treatment; the first group received immunosuppressant alone, the second group received a combination of immunosuppressant and anticoagulant, and the third group was treated with anticoagulation only [7]. Notably, the anticoagulation-alone group received warfarin for a minimum of six months; besides, the immunosuppressant group mostly received azathioprine or others (cyclophosphamide or mycophenolate in case of severe side effects or failure to respond to azathioprine), besides corticosteroids [7]. Recurrence of venous thrombosis was reported in 12.5% of the immunosuppressant group, 5.9% of the combination group, and 75% in the anticoagulation-alone group [7]. Nevertheless, the difference between the immunosuppressant alone and the combination was not statistically significant (p = 0.601) [7]. 

Moreover, although oral anticoagulants are not recommended in the treatment of vascular BD patients, they might be required in the presence of ICTF [5]. Samaniego et al. had concluded in their single-center case-control study that anticoagulation is recommended only for patients with recurrent or refractory thromboembolism with low bleeding risk [6]. On the other hand, a case of neglected and partially treated vascular BD in the form of leg vein thrombosis was found to have RV mass along with pulmonary embolism, pericardial effusion, and complete inferior vena cava occlusion [8]. Subsequently, he was treated with full-dose dalteparin, for which the RV thrombus size was decreased two weeks later, but there was an extension of the pulmonary embolism [8]. Additionally, he was started on high-dose prednisolone, colchicine, prophylactic Bactrim, and cyclophosphamide in continuation with warfarin [8].

In addition, another case of BD developed two intracardiac thrombi while on oral prednisolone and colchicine [9]. He was immediately started on low-molecular-weight heparin followed by oral warfarin in addition to immunosuppressant (prednisolone and monthly cyclophosphamide) [9]. Interestingly, four months later, cardiac MRI revealed complete resolution of the intracardiac, pulmonary, and superior vena cava thrombosis [9]. The latest case report can conclude that prednisolone and colchicine may not be adequate in preventing vascular complications of BD.

While most data in the literature are questioning the role of anticoagulants in vascular BD patients, we believe based on reviewing the literature that, first, anticoagulation must be used in addition to immunosuppressant therapy in cardiac involvement of BD, given that the high mortality linked to an intracardiac thrombus, with caution to the risk of bleeding; secondly, in BD patient with recurrent venous thromboembolism while on immunosuppressants, the addition of anticoagulant might provide therapeutic and prophylactic effect, with caution to the bleeding risk; thirdly, the development of venous thromboembolism in BD patient while on immunosuppressant may require additional immunosuppressant; similar to our case where azathioprine was added to infliximab infusion along with four weeks of oral prednisolone.

## Conclusions

BD is a systemic vasculitis disease involving several body organs, including cardiac involvement, which carries a high mortality rate. Thromboembolism is a well-recognized vascular complication of BD. However, while the standard gold treatment for thromboembolism in BD is immunosuppressant, anticoagulants might provide a beneficial effect in treating and preventing thromboembolism, particularly with intracardiac thrombus co-existence. Nevertheless, multi-center double-blinded randomized clinical trials might not be applicable due to the scarcity of this disease. However, further studies are warranted to establish the efficacy and safety of anticoagulants in addition to immunosuppressants in managing thromboembolisms of BD, especially in the presence of ICTF. 
